# Pediatric acute respiratory distress syndrome in a Moroccan intensive care unit: a retrospective observational study of 23 cases

**DOI:** 10.11604/pamj.2023.44.201.35724

**Published:** 2023-04-26

**Authors:** Said Benlamkaddem, Fatima Bouyerman, Mohamed Adnane Berdai, Mustapha Harandou

**Affiliations:** 1 Pediatric and Maternal Critical Care Unit, Hassan II University Hospital, Fez, Morocco

**Keywords:** Pediatric acute respiratory distress syndrome, lung protective ventilation, prone position

## Abstract

Acute respiratory distress syndrome (ARDS) is a life-threatening condition despite medical development. Unlike adult, ARDS, in pediatric population, has been recently defined in the Pediatric Acute Lung Injury Consensus Conference (PALICC), 2015. We conduct a retrospective descriptive study, in pediatric intensive care unit (PICU) of Hassan II University Hospital during a period of 2 years (2019 to 2021) in which we included 23 pediatric cases of ARDS defined using 2012 Berlin criteria. They represent 2.7% of all patients admitted in our unit (23 patients of 850 admissions), with a male predominance 17 males/6 females, the median of age was 4.6 years-old (2 months to 14 years-old). Pediatric acute respiratory distress syndrome (PARDS) cases were stratified as mild in 13% (n=3), moderate in 52% (n=12), and severe in 35% (n=8). The etiologies were of pulmonary origin (pneumonia, aspiration, pulmonary contusion, and foreign body) in 79% of cases (n=18), and extra-pulmonary origin (sepsis, burn and major trauma) in 21% (n=5). The management was based on lung protective invasive mechanical ventilation (95%, n=22), Prone positioning was applied (26%, n=6), inhaled nitric oxide (iNO) was used in (35%, n=8), recruitment maneuvers (56%, n=13), neuromuscular blockade (NMB) (74%, n=17) and extracorporeal membrane oxygenation (ECMO) in 1 case. The outcome was favorable in 65% (n=15) with a mean PICU-stay of 20 days (SD=16 days). Overall mortality rate was 35% (n=8), and 100% (n=5) in case of extrapulmonary (indirect) etiologies. It was proportional to the disease severity, 50% (4 of 8 cases), 33% (4 of 12 cases), and no death respectively in severe, moderate, and mild PARDS. PARDS in our context is a serious problem as it is more frequent in children < 5 years, a population considered as fragile, with a high mortality rate especially in indirect lung etiologies of PARDS.

## Introduction

Acute respiratory distress syndrome (ARDS) is a life-threatening pulmonary condition that constitutes a major challenge in modern ICU. In ARDS, there is a disruption of alveolar epithelial-endothelial barrier leading to the accumulation of non-cardiogenic pulmonary edema due to inflammation and apoptosis which results in type I respiratory failure characterized by severe hypoxemia. ARDS can be triggered by heterogeneous pulmonary (direct lung injury) or extra-pulmonary (indirect lung injury) etiologies. The primary etiologies are pneumonia, sepsis, aspiration, near drowning, and other clinical conditions respectively.

Acute respiratory distress syndrome (ARDS) was first described by Ashbaugh *et al*. in 1967 in 12 patients series who suffered from severe hypoxemia refractory to oxygen supplements [1]. Multiple definitions were proposed since the first description of ARDS including the 1994 American-European Consensus Conference (AECC) definition [2] and the Berlin definition in 2012 [3]. Although these definitions were set primarily for use in the adult population, until recently, they were also employed in the pediatric setting. Due to the limitations of previous definitions when applied to pediatric population, the Pediatric Acute Lung Injury Consensus Conference (PALICC) published in 2015 a pediatric-specific definition for ARDS [4]. However, mortality remains high in PARDS and in 2020, an investigation conducted by Christina Rufener revealed a failure in PARDS diagnosing which may have impact on PARDS outcome [5].

Unfortunately, there is no specific therapy for PARDS, and the management remains supportive. Primarily therapies include mechanical ventilation that may also aggravate lung injury, sedation, and fluid management. Ancillary therapies include high-frequency ventilation, nitric oxide, recruitment maneuvers, surfactant, steroids, prone positioning, neuromuscular blockades, and finally extracorporeal membrane oxygenation (ECMO). Clinical researches are still on the way to define the best treatment strategy for PARDS. The objective of this retrospective observational study was to describe frequency, demographics, management strategies, and outcome of PARDS in our PICU when we used adult-oriented criteria (2012 Berlin criteria) in the perspective to improve our practices in the context of new international guidelines specific to pediatric population (PALICC, 2015 [4] and PALICC 2, 2023 [6]).

## Methods

**Study design and setting:** this is a retrospective observational monocentric study, conducted in pediatric intensive care unit (PICU) of Hassan II University Hospital, Fez, Morocco, between January 2019 and September 2021.

**Study population:** we evaluated patients aged between 1 month and 15 years with acute respiratory distress syndrome (ARDS) admitted to our Intensive Care Unit (ICU). The diagnosis of ARDS was based on Berlin criteria: hypoxemia within 7 days of a clinical insult; respiratory failure not fully explained by cardiac failure or fluid overload; chest imaging with new infiltrate(s) consistent with pulmonary parenchymal disease; PaO_2_/FiO_2_(P/F) ration ≤ 300 or SpO_2_/FiO_2_(SF) ratio ≤ 264 if on Non-Invasive Ventilation (NIV, oro-nasal mask CPAP ≥ 5 cmH_2_O or BiPAP). Patients with cyanotic heart disease, active perinatal lung disease, within 7 days of cardiopulmonary bypass were excluded from the analysis.

**Procedures:** Berlin 2012 definition was used for the diagnosis of ARDS. The severity stratification, and management were based on ARDS network protocol. Severity of ARDS was defined using PaO_2_/FiO_2_ratio as mild (PaO_2_/FiO_2_between 200 and 300), moderate (PaO_2_/FiO_2_between 100 and 200) and severe (PaO_2_/FiO_2_<100). Protective lung strategy settings were as follow: tidal volume 6 ml/kg of ideal body weight (ideal body weight (IBW)=2*(age in years +4)), plateau pressure < 28 cmH2O, with the objectives of permissive hypoxemia (saturation between 88-95% and PaO_2_: 50-80mmhg), and a permissive hypercapnia (PaCo_2_<55mmhg, and PH>7.25). Positive end-expiratory pressure (PEEP)/Fio_2_ table were used to choose PEEP with a range of 5 and 15 cmH_2_O. Prone positioning was indicated in patients with PaO_2_/FiO_2_< 150, it was applied for duration of 16 hours per day. The indication of using inhaled nitric oxide (iNO) for ARDS was PaO_2_/FiO_2_<100.

**Data collection:** study data were collected retrospectively from both paper charts and electronic medical records of patients using HOSIX electronic data capture tools hosted at Hassan II University Hospital of Fez. Variables collected included history, demographic information, diagnostic parameters, chest imaging results, arterial blood gas analysis, patient comorbidities, therapeutics, and evolution.

**Statistical analysis:** the statistical analysis of the parameters was performed using the SPSS 20 software in the epidemiology laboratory of the Faculty of Medicine and Pharmacy of Fez. Descriptive statistics were used to summarize baseline patient characteristics. The results were expressed in numbers and percentages for the qualitative variables and in means ± standard deviations (SD) for the quantitative variables.

**Ethical considerations:** our study did not require written consent as it was retrospective observational non interventional study. Autonomy and confidentiality have been guaranteed throughout collection and analysis of data.

## Results

**Study population characteristics:** 850 patients were admitted to PICU of university hospital HASSAN II during our study (January 2019 - September 2021). Twenty-three patients were diagnosed with PARDS which represents 2.7% of all admissions. There were 6 female and 17 male patients. The median age was 4.6 years (2 months to 14 years). PARDS is more frequent in infants less than 5 years in our series with a percentage of 73% (n=17) of PARDS cases, 9% (n=2) of patients from 5 to 10 years, and 18% (n=4) of patients aged from 11 years to 14 years (Table 1). The causes of admission were dominated by respiratory distress (52%, n=12), major trauma (22%, n=5), and sepsis (13%, n=3). Three patients presented with comorbidities including 1 case with a cerebral tumor, 1 case with type 1 diabetes mellitus, and 1 case with valvular heart disease. At admission, 60% (n=14) of patients had respiratory failure, 18% (n=4) of patients had a circulatory failure, 13% (n=3) had renal failure, and 30% (n=7) had neurological impairment (Table 1).

**Table 1:** population characteristics

Characteristics	Participant (n=23)
Age, median	4.6 (2 months - 14 years)
2 months to 5 years	17 (73%)
6 to 10 years	2 (9%)
≥ 11 years	4 (18%)
**Gender, n (%)**	
Female	6(27%)
Male	17(73%)
**Comorbidities, n (%)**	
Cerebral tumor	1(4.5%)
Type 1 diabetes	1(4.5%)
Valvular heart disease	1(4.5%)
None	20 (86.5%)
**Causes of admission, n (%)**	
Respiratory distress	12(52%)
Major trauma	5(22%)
Sepsis	3(13%)
Severe burn	1(4.34%)
Diabetic ketoacidosis	1(4.34%)
Status epilepticus	1(4.34%)
**Organ failure at admission, n (%)**	
Respiratory failure	14(60%)
Circulatory failure	4(18%)
Renal failure	3(13%)
Neurological impairment	7(30%)

**Figure 1:**
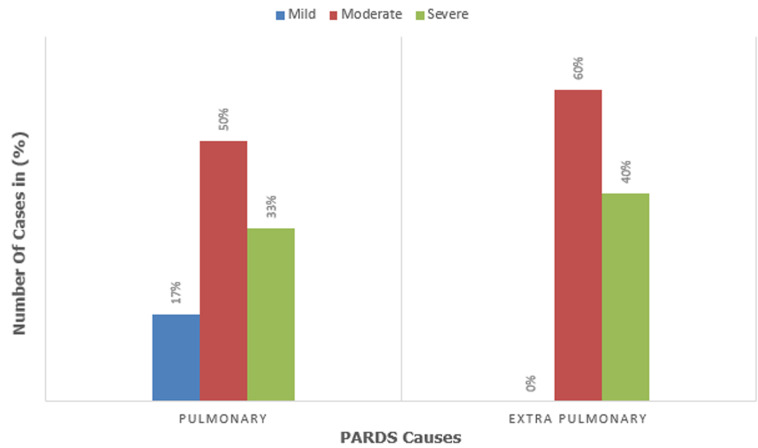
distribution of cases according to direct or indirect cause and severity of illness

**Study PARDS characteristics:** ten patients (43%) were diagnosed with ADRS at admission, and 13 (57%) patients were diagnosed with ADRS during their hospital stay. the etiologies of PARDS were mainly of pulmonary origin (79%, n=18): pneumonia 48% (n=11), aspiration 22% (n=5), lung contusion 4.5% (n=1), and foreign body 4.5% (n=1). The extra-pulmonary causes identified in 22% (n=5) of cases are as follows: sepsis 13% (n=3), major trauma 4.5% (n=1), and severe burn 4.5% (n=1). The severity of illness in our study was stratified as mild in 13% (n=3), moderate in 52% (n=12), and severe in 35% (n=8) (Table 2). PARDS was mild in 17% (3 of 18 cases), moderate in 50% (9 of 18 cases), and severe in 33% (6 of 18 cases) in cases of direct causes. In indirect causes, PARDS was moderate in 60% (3 of 5 cases), and severe in 40% (2 of 5 cases) (Figure 1).

**Table 2:** characteristics of pediatric acute respiratory distress syndrome in our series

Characteristics	Number of patients n (%)
**PARDS diagnosis**	
At admission	10(43%)
At PICU stay	13(57%)
**Causes of PARDS**	
Pulmonary	18 (79%)
Bacterial pneumonia	7 (30%)
Viral pneumonia	4 (18%)
Aspiration	5(22%)
Contusion	1(4.5%)
Foreign object	1(4.5%)
Extra pulmonary	5 (22%)
Sepsis	3 (13%)
Severe burn	1(4.5%)
Major trauma	1(4.5%)
**Risk stratification**	
Mild	3(13%)
Moderate	12(52%)
Severe	8(35%)

PARDS: pediatric acute respiratory distress syndrome; PICU: pediatric intensive care unit

**Management of PARDS:** 95% (n=22) of patients were invasively ventilated, 45% (n=10) of patients were intubated at admission, 32% (n=7) were already on mechanical ventilation, and 22% (n=5) were intubated during hospitalization. The mean duration of invasive ventilation was 13.5 days (SD=9) with extremes ranging between 4 and 30 days. The complications presented in intubated patients were as follows: pneumothorax in 7 patients (30%), atelectasis in 4 patients (17%), and ventilator-associated pneumonia in 13 patients (56%), the principal germs detected was *Acinetobacter baumannii* and *Klebsiella pneumoniae*.

One patient was exclusively treated with non-invasive ventilation (NIV) for a duration of 8 days. Twenty-two patients were systematically under NIV in post-extubation. All patients under mechanical ventilation were sedated using the association of fentanyl and midazolam with the objective of minimal effective sedation. Prone positioning was applied in 6 patients (26% of cases), 1 patient with moderate PARDS, and 5 patients with severe PARDS. iNO was administered to 8 patients (35%) with severe PARDS. Recruitment maneuvers (RM) were performed in 13 patients (56%). Veno-venous extracorporeal membrane oxygenation (ECMO) was used in one patient. Rocuronium was administered in 17 patients (74%), and prednisolone was prescribed to 10 patients (43%) (Table 3).

**Table 3:** management of pediatric acute respiratory distress syndrome

Management	Number of patients n (%)
Invasive ventilation	22(95%)
**Complications**	
Pneumothorax	7(30%)
Atelectasis	4(17%)
Ventilator-associated pneumonia	13(56%)
**Germs**	
*Acinetobacter baumannii*	8(61.5%)
*Klebsiella pneumonia*	4 (30.7%)
*Pseudomonas aeroginosa*	3(23%)
*Serratia marcescens*	1(7.7%)
Polybacterial	5(38.5%)
Non invasive ventilation	1(4.5%)
Prone position	6(26%)
Inhaled oxide nitric (iNO)	8(35%)
Recruitment maneuvers	13(56%)
Veno-venous ECMO	1(4.5%)

ECMO: extracorporeal membrane oxygenation

**Evolution:** the mean PICU stay was 20 days (SD=16 days) ranging between 4 and 51 days. The mortality rate was 35% (n=8) (Table 4), ranging from 17% (3 of 18 cases) in direct (pulmonary) etiologies to 100% (5 of 5 cases) in indirect (extrapulmonary) ones. It was proportional to the disease severity, 50% (4 of 8 cases), 33% (4 of 12 cases), and no death respectively in severe, moderate, and mild PARDS.

**Table 4:** distribution of mortality according to different factors

Factors	Survivors n=15 (65%)	Non-survivors n=8 (35%)
**Gender**		
Male	10 (66.6%)	7(87.5%)
Female	5(33.4%)	1(12.5%)
**Etiologies**		
Direct	15(83%)	3(17%)
Pneumonia	9	2
Aspiration	4	1
Contusion	1	-
Foreign object	1	-
Indirect	-	5(100%)
Sepsis/SIRS	-	3
Severe burn	-	1
Major trauma	-	1
**Severity groups**		
Mild	3 of 3 (100%)	0
Moderate	8 of 12 (67%)	4 of 12 (33%)
Severe	4 of 8 (50%)	4 of 8 (50%)

SIRS: systemic inflammatory response syndrome

## Discussion

This retrospective observational study aimed to describe frequency, demographics, management strategies, and outcome of PARDS in our PICU when we used adult-oriented criteria (2012 Berlin criteria) in the perspective to improve our practices in the context of new international guidelines specific to pediatric population (PALICC, 2015 [4] and PALICC 2, 2023 [6]). The estimated PICU admissions-based incidence of PARDS in our series was 2.7% (23 of 850 admissions) which was equivalent to most reported studies [7,8]. ARDS is more frequent in children aged < 5 years old while the occurrence of ARDS decreases in older children as in our study [9-11]. The elevated frequency of PARDS in children under 5 years may be explained by the immaturity of the immune system along with lung development and maturation that occurs in that range of age. There is a male predominance in all studies including ours [9-11].

Etiologies of PARDS can result from direct causes (i.e. lung injury originating on the alveolar side of the alveolar-capillary membrane) or indirect ones by systemic immune reaction in response to extra pulmonary aggression [8]. In the Paediatric acute respiratory distress syndrome incidence and epidemiology (PARDIE) study, pneumonia presented with 67% and sepsis with 19% of cases were the most common PARDS risk factors. Followed by aspiration 8% and trauma 4% [11]. In our series we noticed predominance of pneumonia in 48% followed by aspiration in 22%, and sepsis in 13%.

Mechanical ventilation is indispensable to ensure adequate gas exchange in patients with acute respiratory failure. However, it may induce lung injury and inflammation (Ventilator-Induced Lung Injury (VILI)) [12]. In 2017, the European Society for Pediatric and Neonatal Intensive Care developed and voted on list of 152 recommendations for mechanical ventilation of critically ill children. Unfortunately, none of the various modes of mechanical ventilation has been demonstrated to improve the outcome in the pediatric population, including patients with PARDS [13]. Lung protective strategy with low tidal volume of 6ml/kg of ideal body weight is the key of ARDS treatment in adult. In PARDS this strategy is not well demonstrated as there is variability of study results [14,15]. Thus, the PALICC group weakly recommends tidal volumes of 3-6 mL/kg for patients with poor compliance, and 5-8 mL/kg for patients with more preserved respiratory compliance [16].

The selection of PEEP is based on the characteristics, extent, and duration of each patient's lung disease. Some tables pairing levels of FIO_2_ and PEEP have been published with a number of randomized ARDS trials. These tables were developed by the investigators to standardize care by using higher PEEP and low systemic oxygen saturation. Most tables are identical to the one used in the ARDS net low tidal volume trial [17].

Because it is often impossible to predict how an individual patient will respond to a lung recruitment attempt, careful individual PEEP titration is the reasonable approach and has been shown to be efficient in terms of improvement of oxygenation and safe in patients with PARDS. There is no consensus on the optimal performance of RMs and no data exist on their effect on mortality, morbidity, length of stay, or duration of mechanical ventilation [18]. RMs were applied on 56% of cases in our study. Unlike many other management strategies in PARDS, a multicenter randomized clinical trial (RCT) evaluating prone positioning in pediatrics is available and demonstrated proning to be safe, but found no difference in duration of mechanical ventilation, mortality, or other health outcomes [19]. The ongoing Prone and Oscillation Pediatric Clinical Trial (PROSpect) study which estimated to accomplish in 2024 hopes to better determine its efficacy in severe PARDS [20]. Prone positioning was performed only in 26% (n=6) of our cases because there were some limitations (severe trauma, severe burn, shock…). A multicenter randomized controlled trial in 2015 found the use of iNO was associated with a significantly reduced duration of mechanical ventilation and significantly greater rate of extracorporeal membrane oxygenation-free survival [21]. The PALICC group did not recommend routine use of iNO [16]. iNO was applied in 8 patients white severe PARDS in our study.

The Pediatric Acute Lung Injury Consensus Conference (PALICC) recommends considering NMB in children with PARDS if sedation alone is inadequate to achieve effective mechanical ventilation, targeting the minimal effective dose [16]. Rocuronium was administered in 17 patients in our study. Recent trials in children failed to show a survival benefit for corticosteroids in ARDS. Consequently, their use is not recommended as routine therapy in PARDS [13,16]. In our study, 10 patients received corticosteroids for pneumonia or sepsis. Mortality rates of PARDS is decreasing over time from 48% to 17% [22]. This may be explained by the adoption of lung protective bundle adapted from adult data in addition to protocolized sepsis care and timely antibiotics. The evolving of definitions for PARDS also contributed into lower mortality rates with the final definition of PALICC group.

Different studies reported different percentages of mortality and the estimated mortality in these studies vary between 17-35% [8]. In PARDIE multicentric cross-sectional study the overall mortality was 17%. It was higher in indirect than in direct lung injury (30% vs 12%) [11]. In our series the mortality rate was 35%. It is lower in PARDS triggered by direct lung injury with 17% (3 cases of 18). There were no survivors in PARDS triggered by indirect lung injury. It should be noted that viral infections such as respiratory syncytial virus and influenza virus are more frequently life-threatening cause of PARDS [23]. A history of prematurity, cancer, left ventricular dysfunction or immune compromise are risk factors for mortality [24].

This study has some limitations including the small sample, the retrospective observational non interventional study design. Despite these limitations, our study allowed us to evaluate our practice and to be compared with other studies. Based on these results, further work using pediatric specific guidelines are ongoing.

## Conclusion

Pediatric acute respiratory distress syndrome (PARDS) is a life-threatening pulmonary condition and one of major challenges in modern PICU and needs to be more explored. In our study the main cause of PARDS was pneumonia. The management was supportive and based on adult-adapted guidelines (lung protective strategy, FiO_2_/PEEP tables, prone positioning, iNO, NMB, and ECMO). Mortality was high, especially in indirect lung causes.

### What is known about this topic

- *Pediatric acute respiratory distress syndrome (PARDS) is a life-threatening condition;*
- *Guidelines from the PALICC are the first in the matter in pediatrics;*
- *Management of PARDS is challenging and is mostly symptomatic*.

### What this study adds

- *We present our experience in PARDS management using adult guidelines;*
- *We highlight this condition of fatality in a fragile population;*
- *This is a support for further studies using PALICC guidelines*.
